# Hernia

**Published:** 1895-02-23

**Authors:** 


### Progress in Surgery.

### HERNIA.

**Strangulated Hernia.**—The statistics of 940 consecutive cases of herniotomy at St. Bartholomew's, St. Thomas's, and Guy's Hospitals, up to 1884[1] show so high a mortality—43 per cent.—that Mr. Croft has collected the records of his herniotomies from May, 1866, to October, 1892,[2] and he has arranged them into two chief groups, viz, those operated on in pre-antiseptic times, and those operated on with antiseptic precautions. In the latter group there are 44 cases with a mortality of 29½ per cent.; in the former the mortality exceeded 50 per cent. This seems enormous, but we must remember, as Mr. Croft points out, that Mr. Treves in 1891 gave the mortality at the London Hospital at nearly 50 per cent. During the last ten years Mr. Bowlby has found that the mortality at St. Bartholomew's has been less than 43 per cent.—only 35·82 per cent., which more nearly approaches Mr. Croft's mortality in his cases operated on during the antiseptic period. Mr. Croft has analysed the causes of death in the 13 cases in which the operation had been performed antiseptically, and he found that nine deaths occurred from exhaustion from protracted sufferings and old age, and that in three of these the gut was gangrenous, and that five were nearly moribund at the time of the operation. There were no deaths from peritonitis. In fact, Mr. Croft's experience is that patients die after herniotomy because the operation is done too late, not in any way from the operation itself, now that peritonitis is so rare after it. Mr. Bowlby[3] considers that the causes of death after herniotomy are exhaustion, from compulsory starvation of several days' duration, and from the vomiting and pain, and that the high mortality is due to the length of time that has elapsed between the onset of the strangulation and the operation. He thinks hardly any patients die from the operation itself, and that in cases operated on in the first twelve hours after strangulation the mortality is trifling, but after three or four days the chances of recovery are but small. Mr. Croft's cases show very clearly that the liability to peritonitis since the introduction of antiseptics into surgery has been greatly lessened. In the pre-antiseptic period several of the patients died of peritonitis and five from erysipelas or pyæmia, but by operating antiseptically Mr. Croft considers we have practically eliminated peritonitis as a cause of death after herniotomy. Bowlby[4] is of the same opinion, for he says that peritonitis or wound complications account only for a small minority of the deaths, and that when peritonitis does occur it usually results from perforation of the bowel, where it was tightly constricted, some days after the operation. Very interesting in connection with Mr. Croft's series of cases at St. Thomas's Hospital are eleven consecutive successful cases of herniotomy performed at the same hospital between September 12th and 22nd of last year. They were all under the care of either Mr. Ballance or Mr. Battle, and are reported[5] to illustrate the success attending the modern method of operation for strangulated hernia, but perhaps they better illustrate the success of the antiseptic operation when the patient happens to come into the hospital early enough, for, as Mr. Bowlby and Mr. Croft point out, the high mortality is due to delay in operating, and they clearly show that if medical men will send the patients into hospital without any delay, the chances of recovery are very good. There was no gangrene or ulceration of the bowel in any, and no adhesion of intestine to the sac. Mr. Battle points out that the essential conditions of success were present, viz., early operation, without too severe or prolonged taxis.

A case reported in the same paper was one where obstruction of the bowels was caused after taxis, by the use of an aperient. Two days after the reduction of a strangulated hernia by taxis a young man took aperient pills. Soon after he began to suffer from great pain in the abdomen and vomited, and the vomiting soon became feculent, with abdominal distension, and collapse. Mr. Ballance performed abdominal section, and found two inches of small intestine paralysed where it had been strangulated, the bowel above being dark red in colour and collapsed below. Professor Ashurst[6] draws attention to what he believes to be the desirable limitation of taxis in cases of strangulated hernia. He thinks that in the hands of an inexperienced practitioner, who sees but few cases of hernia, taxis is an unsafe procedure. His own cases of herniotomy which resulted fatally had been usually subjected to prolonged taxis. The plan of treatment adopted at the Pennsylvania Hospital is, first, to try gentle taxis, and if this does not succeed to apply ice over the hernia and give a moderate dose of opium, and then the hernia often becomes spontaneously reduced or disappears "under the slightest touch." If it does not Ether is given and another attempt made before operation. Mr. Huthe also sounds a note of warning with reference to taxis in strangulated hernia.[7] He fears precious time may be wasted over this manipulation, which is often unsuccessful, and may damage the bowel, and thinks that success will be greatest for those who operate early.

**Gangrenous Hernia.**—Mr. Croft[8] reports thirteen cases of herniotomy for gangrenous hernia occurring in his own practice at St. Thomas's Hospital, and he considers on reviewing them that only two would have borne the operation of primary resection and suture. He believes this operation to be desirable when the patient's condition is good enough to permit of it, but thinks that the question of the advisability of primary resection or the formation of an artificial anus cannot be settled by the mortality following the two operations, for the cause of failure in cases of artificial anus is not in the operation itself but in the conditions which have preceded and accompanied the gangrene. Bowlby pointed out the same fact last year with regard to primary resection, and suggests that the successful series of cases collected by Mr. Kendal Franks, and reported by him to the Medico-Chirurgical Society in March, 1893, were mainly so because they were all early cases of gangrene. Those operated on after the third day of strangulation all died, and he thinks that late resection will be as fatal as the late formation of an artificial anus. The subject was discussed last year at great length before the Chirurgical Society of Paris.[8]

Dr. Chaput communicated two cases of Dr. Martinet's. The first was a case of umbilical hernia, in which the portion of gangrenous bowel was enclosed in a fold of intestine, the edges of which were stitched together by a continuous catgut suture, the gangrenous portion of gut being thus abandoned in the intestinal cavity in the hope that it would be eliminated. Dr. Chaput has discovered records of several other cases treated in the same way. The second case was a successful primary resection for gangrenous femoral hernia, the bowel being united with Lembert's suture. A fæcal fistula formed, but soon closed. He admits that, in referring to the statistics of the mortality of primary resection and the formation of artificial anus respectively, "we must not lose sight of the fact that surgeons reserve the best cases for resection, abandoning the less favourable conditions to artificial anus"; but he quotes the statistics of Mikulicz as favourable to primary resection, as this method only gave a mortality of 33 per cent., whereas with the formation of artificial anus 76 per cent. died. Statistics of all the cases of gangrenous hernia treated by Czerny, Halm, Kocher, Hagedorn, and Mikulicz (118 cases) give a mortality of 59 per cent. Many of these cases were treated by primary resection. Taking them all together, however, and thus cases of all degrees of severity, the death-rate is distinctly lower than the 80 per cent. of deaths in the series of cases published by other surgeons, who have treated all their cases, without exception, by the formation of an artificial anus. This seems really to favour the operation of primary resection as a means of saving life. Dr. Chaput, therefore, prefers primary resection, and would reserve the formation of an artificial anus for cases with great collapse. But he recommends that the resected loop should be maintained for forty-eight hours outside the abdomen between two layers of iodoform gauze, and thus, if leakage occurs, only a temporary fæcal fistula will form, and the peritoneum will not be affected. He agreed with Mikulicz that the hernial ring should always be divided, so as to allow of the thorough irrigation and drainage of the peritoneum. In erosions, or small patches of gangrene, he advises enclosure of the damaged portion by folding in, and union of the peritoneal coat over it. The formation of an artificial anus he thought had, in addition to its disgusting nature, risks of its own, such as abscess of the abdominal wall, or septic thrombosis of the femoral vein, or perforation of the bowel above; but it had the advantage of giving a free outlet for the fæces which might be poisoning the patient. In the discussion which followed the reading of Dr. Chaput's paper, Professor Verneuil, as well as Professor Terrier, and Drs. Segond, Kirmisson, and Lucas-Championnière, objected that intestinal suture was too long an operation for cases with such an amount of prostration as was usually present with gangrenous hernia, but it was suggested that the success which Murphy had attained by means of his button in primary resection might revolutionise the whole question. Dr. Chaput, however, considered that even with circular suture the operation does not last more than half-an-hour in skilful hands. He thought the duration of an operation mattered very little if there was no pain, hæmorrhage, serious traumatism, or prolonged anæsthesia, and intestinal suture could be carried on after the effect of the anæsthetic had passed off, as it was entirely painless, and it was not a severe injury to the bowel or attended with hæmorrhage. Dr. McCosh, of New York, has lately reported[9] three successful cases of primary resection and suture for gangrenous hernia. Two seem to have been in a very prostrate condition, and the third patient was seventy-two years of age. All were successful. The author does not believe in artificial aids, but prefers sutures alone. He considers primary resection and suture the best treatment if the condition of collapse is not too great.

[1] Lancet, 1893, vol. I., p. 1221. [2] American Journal of Medical Science, April 4, 1894. [3] Loc. Cit. [4] Lancet, 1894, vol. II., p. 852 and 911. [5] International Medical Magazine, July, 1894, p. 401. [6] Clinical Journal, May 2, 1894, p. 8. [7] Loc. Cit. [8] Medical Week, 1894, p. 139, 212, 226, 343. [9] Annals of Surgery, June, 1894, and Brit. Med. Journal, 1894, Epitome, July 7.

